# Coarse Food Grains Are Important Actors of Healthy and Sustainable Diets

**DOI:** 10.3390/foods5020025

**Published:** 2016-03-30

**Authors:** Anthony Fardet

**Affiliations:** INRA, Human Nutrition Department, JRU 1019, UNH, CRNH Auvergne, F-63000 Clermont-Ferrand & Clermont Université, Université d'Auvergne, Unité de Nutrition Humaine, BP 10448, F-63000 Clermont-Ferrand, France; anthony.fardet@clermont.inra.fr; Tel.: +33-(0)-473624704

Food health potential is not only due to the sum of its nutrients, but also to its food structure [1]. Food structure confers to foods a matrix effect involving impact on satiety feeling and digestive transit, different nutrient bio-accessibility/bioavailability, a package of bioactive compounds with synergistic physiological effects, and fiber co-passengers [2]. This is why coarse food grains have such an important role to play in human diet. They include cereals/pseudo-cereals, legumes, and nuts and seeds that are carbohydrates-, protein- and lipid-rich, respectively, together with a high density in bioactive protective phytochemicals and micronutrients, and with a generally low glycemic index. When eaten raw (e.g., nuts and seeds) or only minimally-processed (such as muesli), they generally keep their food structure more or less intact for a longer duration during the digestive process. However, the most striking fact is that almost all epidemiological studies reveal protective effects of coarse food grains toward main diet-related chronic diseases, *i.e.*, overweight/obesity, type 2 diabetes, cardiovascular diseases and some cancers [3,4]. Last but not least: if our diets were predominantly based on coarse food grains, this would have a great impact on environment sustainability [5]. Indeed, grain-based foods generally have a low carbon footprint from field to plate [6].

In this Special Issue on “Coarse Food Grain”, focus is on cereal grains, from the point of view of their nutritional composition [7,8], antioxidant properties [9], technological aspects [10] to nutritional impacts in both humans [11] and mice [12]; confirming the high nutritional potential of coarse cereal grains. Van den Broeck *et al.* have focused on oat varieties—that fit into a gluten-free diet of celiac disease patients—and on the presence of health-related compounds and how their levels vary among varieties in response to the type of soil [8]. Their study emphasized the importance of profiling oat varieties with specific compound characteristics. Beloshapka *et al.* have profiled 32 samples from barley, oat, rice and other miscellaneous carbohydrate sources in both coarse and more refined food forms with potential use in pet foods, showing that these cereal ingredients provide a readily available energy source, but also a source of dietary fiber, resistant starch, essential amino acids, and macrominerals [7]. Further, Durazzo *et al.* have studied the antioxidant potential of grains and whole flours of some traditional Italian wheats [9]. They interestingly showed that the distinction between extractable and non-extractable antioxidants is very important for an adequate determination of antioxidant capacity in cereals and represents a key element in the definition of the potential nutritional value of the food matrix under consideration. This was illustrated in the study by Agil *et al.* in mice, aiming at determining the effects of alkyresorcinols extracted from triticale bran and added to a high-fat diet on the development of obesity and oxidative stress [12]. Their results suggest that such a cereal ingredient may be useful as a preventative measure against risks of oxidative damage associated with high-fat diets and obesity. In a further study by Nordlund *et al.,* the disintegration of rye and wheat breads (prepared from refined or wholegrain wheat and rye flours) during *in vitro* gastric digestion has been correlated with glycemic and insulinemic responses in humans [11]. This study emphasizes the important role of cereal food structure on metabolic effect, showing that the insulin response had a negative correlation with the number of larger particles after *in vitro* gastric digestion as well as the amount of soluble fiber and sourdough processing, which could find important application for the diet of diabetic patients. Finally, Giannou *et al.* have focused more on technological aspects of cereal-based foods with the objective of enhancing the quality and sensory characteristics of bread made from frozen dough (from both white and whole-wheat flour), notably improving dough strength and stability during frozen storage by adding vital wheat gluten [10]. Findings from this study indicate that the addition of vital wheat gluten at a level above 2% can significantly improve both white and whole-wheat flour parameters, such as loaf volume, color, texture, and thermal behavior of the samples.

The results of these different studies again illustrated the important role of different whole-grain cereal species as sources of antioxidants and protective bioactive compounds and of their preserved food structure during digestion for preventing chronic disease development. Such a convincing amount of scientific evidence should lead technologists and food scientists to less processed coarse food grains to optimally preserve both the food structure and various bioactive and protective phytochemicals.
